# Terahertz toroidal metasurface biosensor for sensitive distinction of lung cancer cells

**DOI:** 10.1515/nanoph-2021-0520

**Published:** 2021-11-05

**Authors:** Chiben Zhang, Tingjia Xue, Jin Zhang, Longhai Liu, Jianhua Xie, Guangming Wang, Jianquan Yao, Weiren Zhu, Xiaodan Ye

**Affiliations:** Department of Electronic Engineering, Shanghai Jiao Tong University, Shanghai 200240, China; Air and Missile Defense College, Air Force Engineering University, Xi’an, China; Department of Radiology, Shanghai Chest Hospital, Shanghai Jiao Tong University, Shanghai 200030, China; Advantest (China) Co., Ltd, Shanghai 201203, China; College of Precision Instruments and Opto-Electronics Engineering, Institute of Laser and Optoelectronics, Tianjin University, Tianjin 300072, China; Department of Radiology, Shanghai Institute of Medical Imaging, Zhongshan Hospital, Fudan University, 180 Fenglin Road, Shanghai 200032, China

**Keywords:** biosenser, lung cancer, metasurface, terahertz

## Abstract

Lung cancer is the most frequently life-threatening disease and the prominent cause of cancer-related mortality among human beings worldwide, where poor early diagnosis and expensive detection costs are considered as significant reasons. Here, we try to tackle this issue by proposing a novel label-free and low-cost strategy for rapid detection and distinction of lung cancer cells relying on plasmonic toroidal metasurfaces at terahertz frequencies. Three disjoint regions are displayed in identifiable intensity-frequency diagram, which could directly help doctors determine the type of lung cancer cells for clinical treatment. The metasurface is generated by two mirrored gold split ring resonators with subwavelength sizes. When placing analytes on the metasurface, apparent shifts of both the resonance frequency and the resonance depth can be observed in the terahertz transmission spectra. The theoretical sensitivity of the biosensor over the reflective index reaches as high as 485.3 GHz/RIU. Moreover, the proposed metasurface shows high angular stability for oblique incident angle from 0 to 30°, where the maximum resonance frequency shift is less than 0.66% and the maximum transmittance variation keeps below 1.33%. To experimentally verify the sensing strategy, three types of non-small cell lung cancer cells (Calu-1, A427, and 95D) are cultured with different concentrations and their terahertz transmission spectra are measured with the proposed metasurface biosensor. The two-dimensional fingerprint diagram considering both the frequency and transmittance variations of the toroidal resonance dip is obtained, where the curves for different cells are completely separated with each other. This implies that we can directly distinguish the type of the analyte cells and its concentration by only single spectral measurement. We envisage that the proposed strategy has potential for clinical diagnosis and significantly expands the capabilities of plasmonic metamaterials in biological detection.

## 1 Introduction

Lung cancer is the second most widespread cancer that accounts for the highest number of cancer-related deaths worldwide [1, 2]. It could be histologically divided into several types, such as squamous cell carcinomas, adenocarcinomas, small cell lung carcinomas, large cell carcinomas, large cell neuroendocrine carcinomas, sarcomatoid and pleomorphic carcinomas, and several other types, according to the new edition of World Health Organization classification [3]. It is well known that cellular heterogeneity has great effects on cancer prognosis and therapeutic strategy. In a good model of adenocarcinoma, the lepidic and acinar subtypes have better prognosis than the micropapillary and solid ones, which might need to adjust the therapy such as lobectomy and adjuvant chemotherapy [4]. Hematoxylin and eosin-stained histological examination is the current routine pathologic diagnostic method of lung tumors [5, 6]. Light microscopy could be used to differentiate most of the histologic subtypes, while immunohistochemistry and gene sequencing compensate for it, which are performed more common on prognosis predictions and targeted medicine selections [7, 8]. Although those techniques are efficient, they are associated with many limitations such as time consuming, high demand for clinical experience, and restrictive experimental conditions. Therefore, it is still highly demanded to develop a real-time, high-sensitive, and yet simple cell detection method for the evaluation of lung cancer cell lines, which would be extremely convenient for identifying the lung cancer molecular subtypes and their differentiations, so as to help surgeons and oncologists to formulate more precise therapeutic schedules.

Plasmonic metasurfaces [9–12] are artificially structured surfaces with subwavelength meta-atoms, aiming to achieve enhanced modulation of light–matter interaction compared to natural materials. Resulting from extremely enhanced interactions between the analytes and the resonant meta-atoms, metasurfaces show great potential for applications in the field of biosensors, especially for the fast and accurate detection of molecular [13–17]. From a physical point of view, the phenomenon of spectral shift caused by diverse biomolecules can be attributed to changes in factors such as refractive index/permittivity, conductivity/resistance, or size of the analytes. Conventionally, Fano [18–21] or electromagnetically induced transparent (EIT)-like [22–25] type plasmonic metasurfaces are often used to construct sharp resonance dips or peaks, respectively. In such metasurfaces, bright and dark modes are simultaneously excited in meta-atoms with asymmetric structures such as spilt ring resonators (SRRs). More recently, another category of metasurfaces, namely plasmonic toroidal metasurfaces [26–30], have excited a lot of interest owing to the feature of weak free-space coupling and unique light localization, which are crucial for achieving high quality factor (*Q*) response and enhanced light–matter interactions for sensing. For example, toroidal metasurfaces have been reported for detecting antibiotic molecules [31] and envelope proteins [32, 33] in mid-infrared and terahertz regime with excellent performance. However, only the frequency change of the toroidal resonance has been monitored in all these metasurface sensors, which significantly limits their application in practical.

In this paper, we propose a label-free biosensor based on a toroidal plasmonic metasurface with high sensitivity in terahertz regime. Analyzed from two degree of freedom including amplitude variations and frequency shift, three common types of non-small cell lung cancer cells, including Calu-1, A427, and 95D, could be clearly distinguished and detected the cell concentrations. By analyzing the experimental results for the three types of cells, we found that Calu-1 leads to the most obvious changes, whose maximum dip frequency shift increases from 53 to 130 GHz and the dip transmittance compared to the bare metasurface increases by 25.4 and 50.8%, respectively, when the cells concentration grows from 200 cells/mm^2^ to 600 cells/mm^2^. Meanwhile, the toroidal response is investigated in simulation when the analyte varies with refractive index, thickness, and dielectric loss. Specially, the slope of the theoretical sensitivity under 12 μm-thick analyte layer was calculated to 485.3 GHz/RIU (RIU, Refractive Index Unit). Furthermore, high stability of oblique incidence is observed for the toroidal plasmonic metasurface. Such a robust feature will greatly reduce the errors caused by experimental manipulation and expand the possibility for compact sensors.

## 2 Theory and simulation

**Figure 1:**
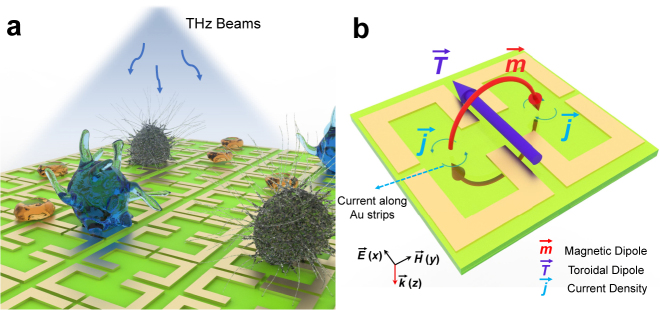
Schematic of the metasurface and meta-atom. (a) Three types of cells cultured on the metasurface are distinguished by the terahertz spectra. (b) Toroidal dipole generated in a pair of asymmetric gold SRRs.

**Figure 2:**
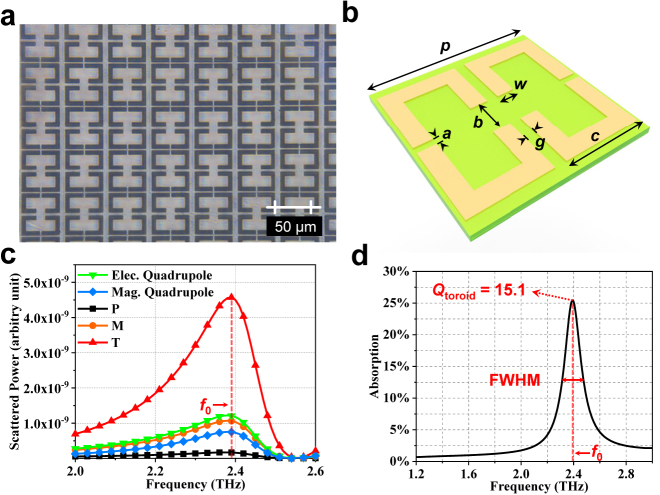
Biosensor sample and toroidal dipole characterization. (a) Micrograph image of the fabricated metasurface biosensor. (b) Unit cell of the metasurface consisting two SRRs on a dielectric slab. The structure parameters are listed as: *p* = 40 μm, *a* = 1.5 μm, *b* = 8 μm, *c* = 18 μm, *g* = 2.5 μm, and *w* = 4 μm. (c) Dispersion of radiation powers for different multipole moments induced in the metasurface. (d) The absorption spectrum of the toroidal metasurface and the calculated *Q* value.

Figure 1a show the schematic view of the terahertz biosensing with a plasmonic toroidal metasurface. Different terahertz transmission spectra could be detected when varying the analytes (lung cancer cells) on the metasurface biosensor, where the weak response with the analytes could be significantly enhanced and captured with accurate statistics for the real-time detection and discrimination of different lung cancer cells. Figure 1b shows the unit cell of the biosensor made of a pair of gold SRRs placed in a symmetrical manner, with the periodicity of 40 μm in the *xoy* plane. The gold (Au) resonators are fabricated on a 500 μm-thick quartz glass substrate (*ɛ* = 3.85), followed by depositing 10 nm titanium (Ti) film and 150 nm Au film with DC conductivity of 4.56 × 10^7^ S/m by magnetron sputtering, as shown in Figure 2a. A photomask with the computer-generated layout was used to scale the pattern of the toroidal metasurface in the Au layer with the help of projection step photolithography. Each of the fabricated samples has an overall size of 5 × 5 mm^2^. When the terahertz beam is normally illuminated with electric filed perpendicular to the SRR gaps, the surface currents (**j**) along the two SRRs are excited in opposite spins, and thus a head-to-tail magnetic field (**m**) is constructed and confined in the unit cell. Such a loop magnetic field results in a toroidal dipole vector along the *x*-axis, shown as the purple arrow in Figure 1b. The toroidal dipole cannot be easily considered as the function of electric or magnetic multipoles while they are produced by the currents that flow along the meridians of torus [28]. The unique characteristics of localization for the toroidal resonance creates weak free-space coupling, which contributes to low-loss and enhanced sensitivity.

To further understand the resonance mechanism of the plasmonic toroidal metasurface, the multipole expansion of the scattered power (*I*) is calculated based on Taylor expansion of the charge and current densities, including the contributions of electric and magnetic multipoles, as well as toroidal dipole [26]:

(1) (1) $$\begin{array}{ll} I & =\frac{2\omega^{4}}{3c^{3}}{\left|P\right|}^{2}+\frac{2\omega^{4}}{3c^{3}}{\left|M\right|}^{2}+\frac{4\omega^{5}}{3c^{4}}{\left|P\cdot T\right|}^{2}\\ & \;+\;\;\frac{2\omega^{6}}{3c^{5}}{\left|T\right|}^{2}+\frac{\omega^{6}}{5c^{5}}\sum {\left|Q_{\alpha \beta }\right|}^{2}+\frac{\omega^{6}}{20c^{5}}\sum {\left|M_{\alpha \beta }\right|}^{2}\\ & \;+\;\;O\left(\frac{1}{c^{5}}\right) \end{array}$$

where *c* is the speed of light, *ω* is the angular frequency, and *α* and *β* represent the coordinates *x*, *y*, or *z*. Here, the multipoles **P**, **M**, **T**, *Q*_*αβ*_, and *M*_*αβ*_ can be expressed as follows:

electric dipole

$$P=\frac{1}{i\omega }\int j{\text{d}}^{3}r,$$

magnetic dipole

$$M=\frac{1}{2c}\int \left(r\times j\right){\text{d}}^{3}r,$$

toroidal dipole

$$T=\frac{1}{10c}\int \left[\left(r\cdot j\right)r-2r^{2}j\right]{\text{d}}^{3}r,$$

electric quadrupole

$$Q_{\alpha \beta }=\frac{1}{i\omega }\int \left[r_{\alpha }j_{\beta }+r_{\beta }j_{\alpha }-\frac{2}{3}\left(r\cdot j\right)\right]{\text{d}}^{3}r,$$

and magnetic quadrupole

$$M_{\alpha \beta }=\frac{1}{3c}\int \left[{\left(r\cdot j\right)}_{\alpha }r_{\beta }+{\left(r\cdot j\right)}_{\beta }r_{\alpha }\right]{\text{d}}^{3}r,$$

where **j** and **r** are the current density and the displacement vectors, respectively. The dispersions of the scattered powers for various multipole moments are shown in Figure 2c, calculated by Matlab programs and *LiveLink for Matlab* of Comsol Multiphysics. It is found that the contribution of the toroidal dipole moment reaches its maximum near 2.39 THz and is ∼4.3 times of **M**, ∼3.8 times of *Q*_*αβ*_, ∼6.1 times of *M*_*αβ*_, and even 27 times of **P**. It is clear that the scattered power oscillated by the proposed SRRs is dominated by the toroidal response, where the power radiated by the other multipoles is significantly suppressed. Note that the electrical quadrupole excitation is the second highest component, which is due to the presence of the four separated gold bars in the meta-atom. The quality (*Q*) factor of the plasmonic toroidal resonator could be estimated by *Q* = *f*_0_/Δ*f* ≈ 15.2, where Δ*f* is the full width at half maximum (FWHM) of the absorption spectrum and *f*_0_ is the resonance frequency [34, 35], as shown in Figure 2d. This *Q* factor is much higher than the one reported in [28] with similar dip magnitude, which would be extremely beneficial for sensing applications.

**Figure 3:**
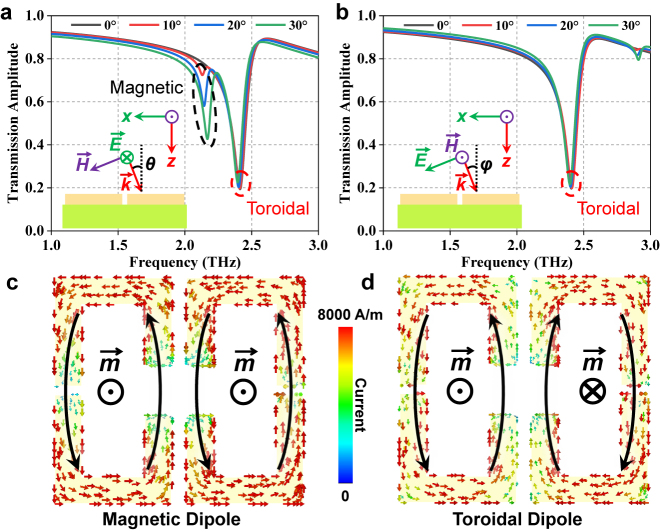
Transmission spectra of the toroidal metasurface under oblique illumination for (a) transverse electric and (b) transverse magnetic modes. Simulated surface currents for (c) magnetic and (d) toroidal resonances at 2.17 THz and 2.39 THz, respectively.

Terahertz metasurface biosensors are typically designed for normal incidence of the excitation beam. It is known that the electromagnetic behavior of a metasurface is commonly sensitive to the direction of the excitation beam, which is crucial important for sensing applications, where additional errors may be introduced in the measurement. However, such an effect in metasurface biosensors has not been evaluated yet. In Figure 3a and b, we show the variation of the transmission spectra of the proposed toroidal metasurface under different oblique angles of incidence, where *θ* and *φ* represent the two oblique angles with respect to the *z*-axis in the two orthogonal planes. When *θ* and *φ* increase from 0 to 30°, the toroidal resonance dip only has tiny shifts both in amplitude and frequency. Compared to the normal incidence case, when varying *θ* (*φ*), the maximum dip frequency shift is only 0.42% (0.66%), whiles the maximum dip amplitude shift is 0.78% (1.33%). The high angular stability allows reasonable operating tolerance to reduce the experimental errors, which will facilitate the development of meta-sensors for portability and industrialization. Besides, in Figure 3a, we see that another resonance is excited at a lower frequency and its resonance strength enhances with increasing *θ*. By observing the current distribution at 2.17 THz for the case *θ* = 30° in Figure 3c, it is seen that two loop current paths with same spin direction occurred on the surface of the two SRRs owing to the introduction of a magnetic field component along with *z*-axis, which is consistent with the characteristic of a magnetic dipole. On the contrary, the toroidal dipole could be recognized at 2.39 THz by the opposite spin currents, as shown in Figure 3d.

**Figure 4:**
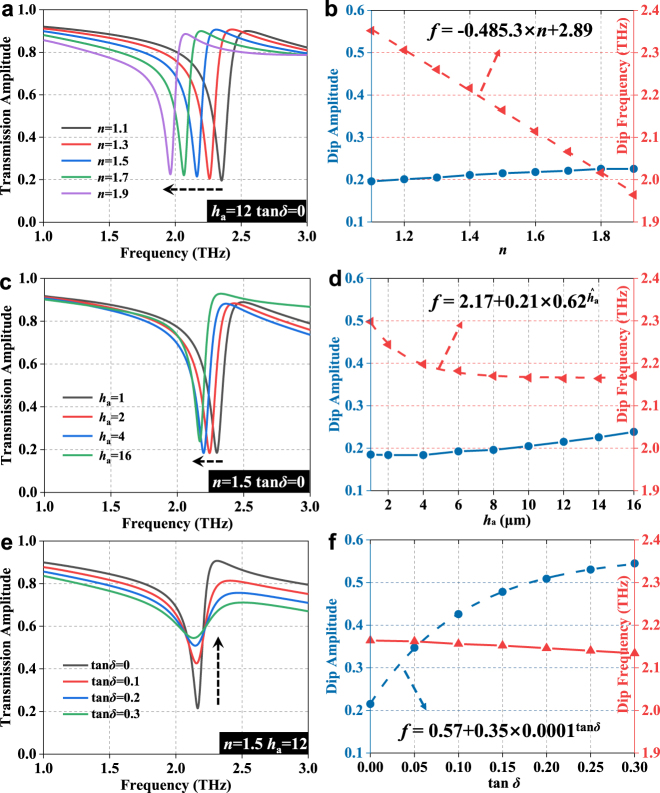
Transmission spectra with the variation of (a) refractive index, (b) height, and (c) dielectric loss of the analyte, where the variations of the corresponding amplitude and frequency of the resonance dip are shown in (d), (e), and (f), respectively. Each parameter varies while the other two remain fixed. Here, *h*_a_ is nondimensionalized by ${\hat{h}}_{\text{a}}=h_{\text{a}}$ /1 μm.

To further investigate the performance of the plasmonic toroidal metasurface for sensing application, we numerically study the transmission spectra of the metasurface when covered by analytes with different properties (parameters), including refractive index *n*, height *h*_a_, and loss tangent tan *δ*. The simulated investigations here all follow the controlled variable criterion. Firstly, the sensitivity is examined from the amplitude and frequency of resonance dip by varying the refractive index from 1.1 to 1.9. As shown in Figure 4a, the sharp toroidal resonance shows apparently red shift while the dip transmittance keeps almost unchanged with the increase of the refractive index. When the analyte with higher permittivity is attached onto the surface of the meta-structure, the resonant frequency would shift to lower frequencies. Based on the trend of resonance dip frequency shift with refractive index, this relationship can be a linear fit, as shown in Figure 4d, where the slope of the line, namely sensitivity (*S*_n_) for refractive index, is as high as 485.3 GHz/RIU, which is higher than those reported in [21, 22]. Such a high sensitivity is raised by the strong interaction at near field produced by the toroidal plasmonic resonance. Corresponding to lung cancer cells, the equivalent refractive index depends on the composition of the cells and the number and sparseness of the organelles within it.

Then, we study the impact of the height as another essential property of the analyte. Figure 4c shows the variation of the resonance spectrum, which also occurs redshift when increasing the thickness of analyte. When *n* is set as 1.5 and the dielectric loss is ignored, we found that the dip frequency shift gradually slows down and saturates later. The fitted shift curve could be obtained as $f=2.17+0.21\times 0.62^{h_{\text{a}}}$ by an exponential function, which has good agreement with the simulation results, as shown in Figure 4d. We find that the resonance dip frequency has significant variations with minor height and it will reach saturation value of 2.17 THz when the height of analyte larger than 12 μm. This is due to the fact that the strong electric field exists only in the localized region above the metasurface and weakens exponentially with the increasing distance. On the contrary, Figure 4d shows significant changes of the resonance dip amplitude at larger thicknesses (higher than 8 μm) rather than when the analyte is thin. Hence, the strategy of observing both changes in amplitude and frequency can effectively characterize the distinction owing to different heights, for cell relevance to size.

At last, the dielectric loss in the analyte also leads to distinct response in the transmission spectrum of the toroidal biosensor. Dielectric dissipation factor (tan *δ*) is usually regarded as a quantitative parameter to describe the dielectric loss as a critical index. As shown in Figure 4e, the resonance dip gradually weakens as tan *δ* increases from 0 to 0.3. This means that the *Q* factor gradually decreases due to the decrease of the energy storage efficiency, since partial energy is dissipated in the lossy media. Of course, since the loss cannot be increased indefinitely for common conducting medium including cells, this deterioration of resonant dip is also limited to some extent, as indicated by the fitted curve in Figure 4f. Nevertheless, when tan *δ* is less than 0.3, the change of the dip amplitude is significant, which facilitates the distinction between analytes with different dielectric losses. Besides, the dielectric loss has a minor effect on the frequency of the resonance dip, as shown in Figure 4f. Therefore, the composition of lung cancer cells can indirectly lead to differences in losses, so that changes in the transmission spectrum regarding amplitude can be captured by us.

## 3 Experiment and results

**Figure 5:**
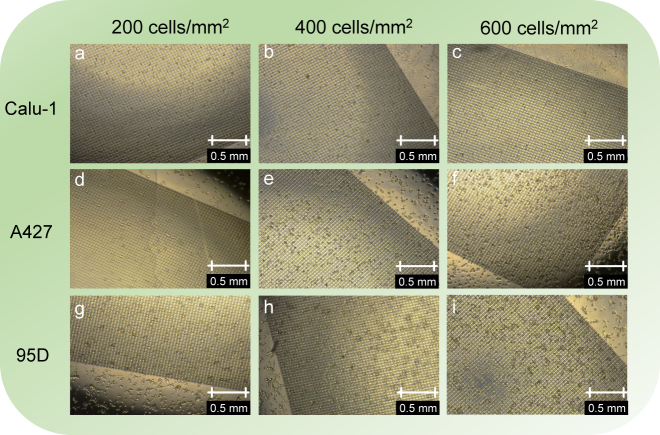
Three types of lung cancer cells Calu-1 (a–c), A427 (d–f), 95D (g–i) with different concentration of 200 cells/mm^2^, 400 cells/mm^2^, and 600 cells/mm^2^ cultured on toroidal plasmonic metasurface.

In our experiment, three types of lung cancer cells are cultured on the surface of plasmonic metasurface samples to verify the practical performance for cell detection and distinction. The three types of lung cancer cells, all acquired from the Central Laboratory of Shanghai Chest Hospital, were cultured in Roswell Park Memorial Institute (RPMI) 1640 medium (Sigma) containing 10% fetal bovine serum (Gibco), 100 IU/ml penicillin, and 100 μg/ml streptomycin (Procell) at 37 °C with 5% CO_2_ in a humidified atmosphere. The adherent cells in the logarithmic growth phase were collected after a series of process including digesting with 0.25% trypsin ethylenediaminetetraacetic acid (EDTA) solution (keyGEN) and centrifugal at 125 × *g* for 5 min. The cell numbers were then counted in order to resuspend the cells to a certain concentration with fresh medium. For improving the utilization of cells, we designed a short round acrylic tube with an inner diameter of 8 mm to place on the quartz substrate and barely reveal the biosensor in the central position. Acrylic tubes were sterilized and placed in 6-well plates and vaseline was smeared outside of the tube-biosensor junction to ensure the watertightness. The preplanned cell suspension of certain concentration was seeded on the surface of the biosensor and cultured again in the environment mentioned above. The micrographs of the fabricated biosensor cultured by Calu-1, A427, and 95D cells with gradient concentrations including 200 cells/mm^2^, 400 cells/mm^2^, and 600 cells/mm^2^, are shown in Figure 5a–i. Before measurement, the fabricated biosensor was washed twice gently with phosphate-buffered saline (PBS) solution (Beyotime), with excess moisture on the surface soaked up by blotting papers.

**Figure 6:**
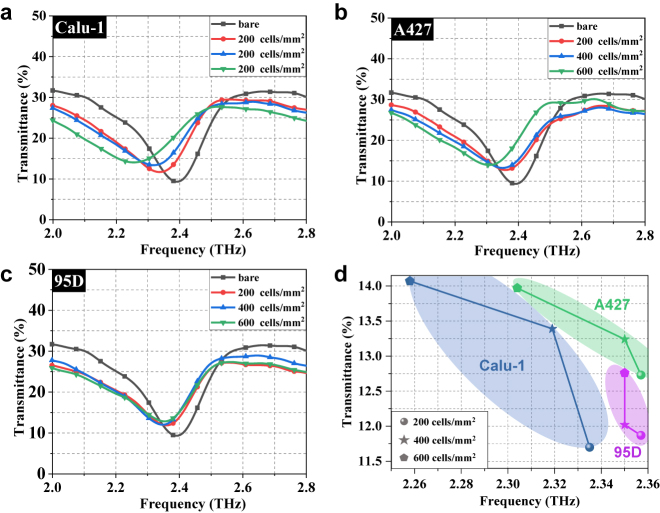
The measured transmission spectra of the proposed biosensor covered by three types of lung cells including (a) Calu-1, (b) A427, and (c) 95D with concentrations of 200 cells/mm^2^, 400 cells/mm^2^, and 600 cells/mm^2^. (d) The shifts of frequency and transmittance of the resonance dip as the cellular concentration changes for Calu-1 (blue), A427 (green), and 95D (violet).

In experiments, a standard terahertz time-domain spectroscopy (Advantest TAS7500SU) is used to measure the transmission spectrum of the samples. All three types of cells, Calu-1, A427, and 95D, grow adherently on the surfaces of the metasurface biosensors. Their terahertz transmittance spectra with different concentrations are plotted in Figure 6a–c. In order to compare the effect of the cells on the toroidal resonance, the spectral curve of the bare biosensor is also plotted. The attachments of all three types of cells cause varying degrees of redshifts for dip frequency and increases for dip transmittance, which is consistent with our simulation results on the assumed analytes. It is seen that the most significant change is found in the case of Calu-1 type cells. With the cell concentration increasing, the dip frequency redshifts are 53 GHz, 69 GHz, and 130 GHz with respect to the bare case, for the cell concentrations of 200 cells/mm^2^, 400 cells/mm^2^, and 600 cells/mm^2^, respectively. Meanwhile, noticeable variations of the dip transmittance for the corresponding concentrations are 2.37%, 4.06%, and 4.74%, which increase by 25.4%, 43.5%, and 50.8% as compared to the bare case, when the cells concentration grows from 200 cells/mm^2^, 400 cells/mm^2^, to 600 cells/mm^2^, respectively. On another hand, for the A427 cells, the maximum variations of transmittance and frequency decrease to be 84 GHz and 4.64%, respectively. The reason for this distinction is attributed to the different physical properties of the two cells in terms of refractive index, size, and dielectric loss, etc., which may be also related to the water content and karyoplasmic ratio of the organism itself. Compared to the two aforementioned cells, the transmission spectrum of 95D is more individuation. Its frequency and transmittance shifts of resonance dip are very tiny that only about 8 GHz and 3.43%, respectively.

Further, according to the variation of the resonance dip in terms of both amplitude and frequency, the characteristic fingerprints of lung cells in the terahertz regime was obtained at different concentrations, as shown in Figure 6d. Three completely separated regions are clearly seen for Calu-1 (blue), A427 (green), and 95D (violet). Even though the amplitude and frequency of the resonance dip do not change linearly respected to the cell concentrations, these trends are basically monotonic and the two-dimensional characteristic regions of different cells are completely separate with each other, as shown in Figure 6d. Therefore, we can directly determine the region (cell type) it belongs to in the two-dimensional intensity-frequency diagram. Such a metasurface-based biosensor could be a great potential for real time and accurate molecule recognition of lung cells, offering a significant step toward practical clinical applications.

## 4 Conclusions

In summary, we have demonstrated a label-free biosensing technology for detection and distinction of lung cancer cells owing to the advantage of strong electromagnetic field confinement by the toroidal plasmonic resonance. A low-loss toroidal metasurface is proposed with high sensitivity of 485.3 GHz/RIU. Here, we would like to note that the *Q* factor of the proposed metasurface sensor is not significantly high. This could be improved by using an all dielectric counterpart [36, 37], where a much sharper resonance and correspondingly higher *Q* value could be achieved, owing to the elimination of ohmic losses naturally available in metal structures. Additionally, the robust angular stability of the toroidal metasurface biosensor is emphasized with a maximum frequency shift less than 0.66% related to center frequency and a maximum transmittance shift less than 1.33%, which would be helpful to reduce experimental errors and make potential progress for practical clinical applications. To experimentally verify the excellent sensing performance, three types of lung cancer cells are cultured on the surface of metasurface biosensor with different cell concentrations. From the measured transmission spectra, we find that the three types of lung cancer cells, Calu-1, A427, and 95D, have remarked differences in terms of transmittance and frequency shifts and predictable trends in the resonance dip with concentration. The fingerprint spectrum of the lung cancer cells can be drawn according to the individual differences of the resonance spectra, which is characterized in terms of both transmittance and frequency. The proposed metasurface sensor could offer a powerful tool to directly distinguish the type of the analyte cells and its concentration with only single measurement. Therefore, we envision that the proposed label-free, robust, real-time strategy based on toroidal metasurface possesses bright prospective to be employed for early lung cancer detection and modern biosensing applications.
